# Bioavailability, distribution and clearance of tracheally-instilled and gavaged uncoated or silica-coated zinc oxide nanoparticles

**DOI:** 10.1186/s12989-014-0044-6

**Published:** 2014-09-03

**Authors:** Nagarjun V Konduru, Kimberly L Murdaugh, Georgios A Sotiriou, Thomas C Donaghey, Philip Demokritou, Joseph D Brain, Ramon M Molina

**Affiliations:** Center for Nanotechnology and Nanotoxicology, Molecular and Integrative Physiological Sciences Program, Department of Environmental Health, School of Public Health, Harvard University, 665 Huntington Avenue, Boston, 02115 MA USA

**Keywords:** Zinc oxide, Nanoparticles, Pharmacokinetics, Bioavailability, Silica coating, Nanotoxicology

## Abstract

**Background:**

Nanoparticle pharmacokinetics and biological effects are influenced by several factors. We assessed the effects of amorphous SiO_2_ coating on the pharmacokinetics of zinc oxide nanoparticles (ZnO NPs) following intratracheal (IT) instillation and gavage in rats.

**Methods:**

Uncoated and SiO_2_-coated ZnO NPs were neutron-activated and IT-instilled at 1 mg/kg or gavaged at 5 mg/kg. Rats were followed over 28 days post-IT, and over 7 days post-gavage. Tissue samples were analyzed for ^65^Zn radioactivity. Pulmonary responses to instilled NPs were also evaluated at 24 hours.

**Results:**

SiO_2_-coated ZnO elicited significantly higher inflammatory responses than uncoated NPs. Pulmonary clearance of both ^65^ZnO NPs was biphasic with a rapid initial t_1/2_ (0.2 - 0.3 hours), and a slower terminal t_1/2_ of 1.2 days (SiO_2_-coated ZnO) and 1.7 days (ZnO). Both NPs were almost completely cleared by day 7 (>98%). With IT-instilled ^65^ZnO NPs, significantly more ^65^Zn was found in skeletal muscle, liver, skin, kidneys, cecum and blood on day 2 in uncoated than SiO_2_-coated NPs. By 28 days, extrapulmonary levels of ^65^Zn from both NPs significantly decreased. However, ^65^Zn levels in skeletal muscle, skin and blood remained higher from uncoated NPs. Interestingly, ^65^Zn levels in bone marrow and thoracic lymph nodes were higher from coated ^65^ZnO NPs. More ^65^Zn was excreted in the urine from rats instilled with SiO_2_-coated ^65^ZnO NPs. After 7 days post-gavage, only 7.4% (uncoated) and 6.7% (coated) of ^65^Zn dose were measured in all tissues combined. As with instilled NPs, after gavage significantly more ^65^Zn was measured in skeletal muscle from uncoated NPs and less in thoracic lymph nodes. More ^65^Zn was excreted in the urine and feces with coated than uncoated ^65^ZnO NPs. However, over 95% of the total dose of both NPs was eliminated in the feces by day 7.

**Conclusions:**

Although SiO_2_-coated ZnO NPs were more inflammogenic, the overall lung clearance rate was not affected. However, SiO_2_ coating altered the tissue distribution of ^65^Zn in some extrapulmonary tissues. For both IT instillation and gavage administration, SiO_2_ coating enhanced transport of ^65^Zn to thoracic lymph nodes and decreased transport to the skeletal muscle.

**Electronic supplementary material:**

The online version of this article (doi:10.1186/s12989-014-0044-6) contains supplementary material, which is available to authorized users.

## Background

Zinc oxide nanoparticles (ZnO NPs) are widely used in consumer products, including ceramics, cosmetics, plastics, sealants, toners and foods [1]. They are a common component in a range of technologies, including sensors, light emitting diodes, and solar cells due to their semiconducting and optical properties [2]. ZnO NPs filter both UV-A and UV-B radiation but remain transparent in the visible spectrum [3]. For this reason, ZnO NPs are commonly added to sunscreens [4] and other cosmetic products. Furthermore, advanced technologies have made the large-scale production of ZnO NPs possible [5]. Health concerns have been raised due to the growing evidence of the potential toxicity of ZnO NPs. Reduced pulmonary function in humans was observed 24 hours after inhalation of ultrafine (<100 nm) ZnO [6]. It has also been shown to cause DNA damage in HepG2 cells and neurotoxicity due to the formation of reactive oxygen species (ROS) [7],[8]. Recently, others and we have demonstrated that ZnO NPs can cause DNA damage in TK6 and H9T3 cells [9],[10]. ZnO NPs dissolve in aqueous solutions, releasing Zn^2+^ ions that may in turn cause cytotoxicity and DNA damage to cells [9],[11]–[13].

Studies have shown that changing the surface characteristics of certain NPs may alter the biologic responses of cells [14],[15]. Developing strategies to reduce the toxicity of ZnO NPs without changing their core properties (safer-by-design approach) is an active area of research. Xia et al. [16] showed that doping ZnO NPs with iron could reduce the rate of ZnO dissolution and the toxic effects in zebra fish embryos and rat and mouse lungs [16]. We also showed that encapsulation of ZnO NPs with amorphous SiO_2_ reduced the dissolution of Zn^2+^ ions in biological media, and reduced cell cytotoxicity and DNA damage in vitro [17]. Surface characteristics of NPs, such as their chemical and molecular structure, influence their pharmacokinetic behavior [18]–[20]. Surface chemistry influences the adsorption of phospholipids, proteins and other components of lung surfactants in the formation of a particle corona, which may regulate the overall nanoparticle pharmacokinetics and biological responses [19]. Coronas have been shown to influence the dynamics of cellular uptake, localization, biodistribution, and biological effects of NPs [21],[22].

Coating of NPs with amorphous silica is a promising technique to enhance colloidal stability and biocompatibility for theranostics [23],[24]. A recent study by Chen et al. showed that coating gold nanorods with silica can amplify the photoacoustic response without altering optical absorption [25]. Furthermore, coating magnetic NPs with amorphous silica enhances particle stability and reduces its cytotoxicity in a human bronchial epithelium cell line model [26]. Amorphous SiO_2_ is generally considered relatively biologically inert [27], and is commonly used in cosmetic and personal care products, and as a negative control in some nanoparticle toxicity screening assays [28]. However, Napierska et al. demonstrated the size-dependent cytotoxic effects of amorphous silica *in vitro*[29]. They concluded that the surface area of amorphous silica is an important determinant of cytotoxicity. An in vivo study using a rat model demonstrated that the pulmonary toxicity and inflammatory responses to amorphous silica are transient [30]. Moreover, SiO_2_-coated nanoceria induced minimal lung injury and inflammation [31]. It has also been demonstrated that SiO_2_ coating improves nanoparticle biocompatibility in vitro for a variety of nanomaterials, including Ag [32], Y_2_O_3_[33], and ZnO [17]. We have recently developed methods for the gas-phase synthesis of metal and metal oxide NPs by a modified flame spray pyrolysis (FSP) reactor. Coating metal oxide NPs with amorphous SiO_2_ involves the encapsulation of the core NPs in flight with a nanothin amorphous SiO_2_ layer [34]. An important advantage of flame-made NPs is their high purity. Flame synthesis is a high-temperature process that leaves no organic contamination on the particle surface. Furthermore, the presence of SiO_2_ does not influence the optoelectronic properties of the core ZnO nanorods. Thus, they retain their desired high transparency in the visible spectrum and UV absorption rendering them suitable for UV blocking applications [17]. The SiO_2_ coating has been demonstrated to reduce ZnO nanorod toxicity by mitigating their dissolution and generation of ions in solutions, and by preventing the immediate contact between the core particle and mammalian cells. For ZnO NPs, such a hermetic SiO_2_ coating reduces ZnO dissolution while preserving the optical properties and band-gap energy of the ZnO core [17].

Studies examining nanoparticle structure-pharmacokinetic relationships have established that plasma protein binding profiles correlate with circulation half-lives [27]. However, studies evaluating the relationship between surface modifications, lung clearance kinetics, and pulmonary effects are lacking. Thus, we sought to study the effects of amorphous SiO_2_ coating on ZnO pulmonary effects and on pharmacokinetics of ^65^Zn when radioactive ^65^ZnO and SiO_2_-coated ^65^ZnO nanorods are administered by intratracheal instillation (IT) and gavage. We explored how the SiO_2_ coating affected acute toxicity and inflammatory responses in the lungs, as well as ^65^Zn clearance and tissue distribution after IT instillation over a period of 28 days. The translocation of the ^65^Zn from the stomach to other organs was also quantified for up to 7 days after gavage. Finally, we examined how the SiO_2_ coating affected the urinary and fecal excretion of ^65^Zn during the entire observation period.

## Results

### Synthesis and characterization of ZnO and SiO_2_-coated ZnO NPs

**Figure 1:**
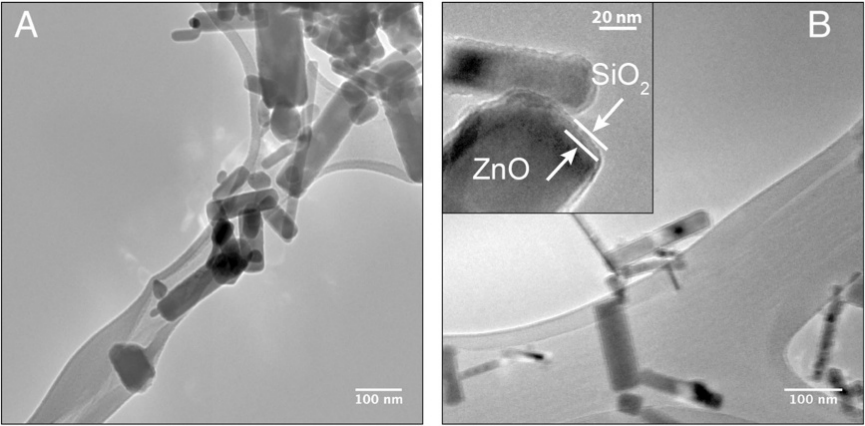
**Physicochemical characterization of test materials.** Transmission electron micrograph of uncoated ZnO **(A)** and SiO_2_-coated ZnO **(B)** NPs. The thin silica coating of approximately 5 nm is shown in B, inset.

Uncoated and SiO_2_-coated ZnO NPs were made by flame spray pyrolysis using the Versatile Engineered Nanomaterial Generation System at Harvard University [35],[17]. The detailed physicochemical and morphological characterization of these NPs was reported earlier [36],[17]. The ZnO primary NPs had a rod-like shape with an aspect ratio of 2:1 to 8:1 (Figure 1) [37],[17]. Flame-made nanoparticles typically exhibit a lognormal size distribution with a geometric standard deviation of σ_**g**_ **=** 1.45 [38]. To create the SiO_2_-coated ZnO nanorods, a nanothin (~4.6 ± 2.5 nm) amorphous SiO_2_ layer encapsulated the ZnO core [17] (Figure 1B). The amorphous nature of the silica coating was verified by X-ray diffraction (XRD) and electron microscopy analyses [17]. The average crystal size of uncoated and SiO_2_-coated NPs were 29 and 28 nm, respectively [39]. Their specific surface areas (SSA) were 41 m^2^/g (uncoated) and 55 m^2^/g (SiO_2_-coated) [40]. The lower density of SiO_2_ compared to ZnO contributes to the higher SSA of the SiO_2_-coated ZnO than uncoated NPs. The extent of the SiO_2_ coating was assessed by X-ray photoelectron spectroscopy and photocatalytic experiments. These data showed that less than 5% of ZnO NPs were uncoated, as some of the freshly-formed core ZnO NPs may escape the coating process [41],[17]. Furthermore, the ZnO dissolution of the SiO_2_-coated nanorods was significantly lower than the uncoated NPs in culture medium over 24 h [17]. The Zn^2+^ ion concentration reached equilibrium after 6 hours for the coated NPs (~20%), while the uncoated ones dissolved at a constant rate up to 24 hours [17]. For both IT and gavage routes, the NPs were dispersed in deionized water by sonication at 242 J/ml. The hydrodynamic diameters were 165 ± 3 nm (SiO_2_-coated) and 221 ± 3 nm (uncoated). The zeta potential values in these suspensions were 23 ± 0.4 mV (uncoated) and −16.2 ± 1.2 (SiO_2_-coated). The zeta potential differences between these two types of NPs were observed at a pH range of 2.5-8.0 [17], which includes the pH conditions in the airways/alveoli and small and large intestines. The post-irradiation hydrodynamic diameter and zeta potential in water suspension were similar to those of pristine NPs used in the lung toxicity/inflammation experiments.

### Pulmonary responses to intratracheally instilled ZnO and SiO_2_-coated ZnO

**Figure 2:**
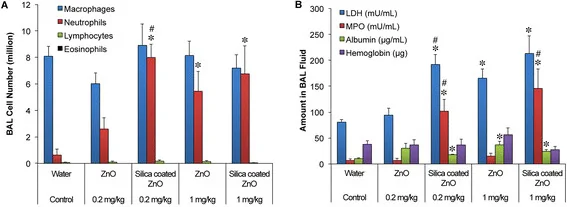
**Cellular and biochemical parameters of lung injury and inflammation in bronchoalveolar lavage (BAL).** Tracheally instilled ZnO and SiO_2_-coated ZnO induced a dose-dependent lung injury and inflammation at 24 hours. **(A)** Significant increases in BAL neutrophils were observed at 1 mg/kg of both NPs (n = 6/group). At the lower dose of 0.2 mg/kg (n = 4-6/group), only the SiO_2_-coated ZnO (n = 4) induced significant neutrophil influx in the lungs. **(B)** Similarly, significant increases in LDH, myeloperoxidase and albumin were observed at 1 mg/kg of both NPs, and at 0.2 mg of SiO_2_-coated ZnO. (*P < 0.05, vs. control, #P < 0.05, SiO_2_-coated ZnO versus ZnO).

We compared the pulmonary responses to uncoated versus SiO_2_-coated ZnO NPs at 24 hours after IT instillation in rats. Groups of 4–6 rats received 0, 0.2 or 1 mg/kg of either type of NP. We found that IT-instilled coated and uncoated ZnO NPs induced a dose-dependent injury and inflammation evident by increased neutrophils, elevated levels of myeloperoxidase (MPO), albumin and lactate dehydrogenase (LDH) in the bronchoalveolar lavage (BAL) fluid at 24 hours post-instillation (Figure 2). At the lower dose of 0.2 mg/kg, only the SiO_2_-coated ZnO instilled rats (n = 4) showed elevated neutrophils, LDH, MPO, and albumin levels. But at 1 mg/kg, both types of NPs induced injury and inflammation to the same extent, except that MPO was higher in rats instilled with SiO_2_-coated ZnO NPs.

### Pharmacokinetics of intratracheally-instilled uncoated or SiO_2_-coated ^65^ZnO NPs

**Figure 3:**
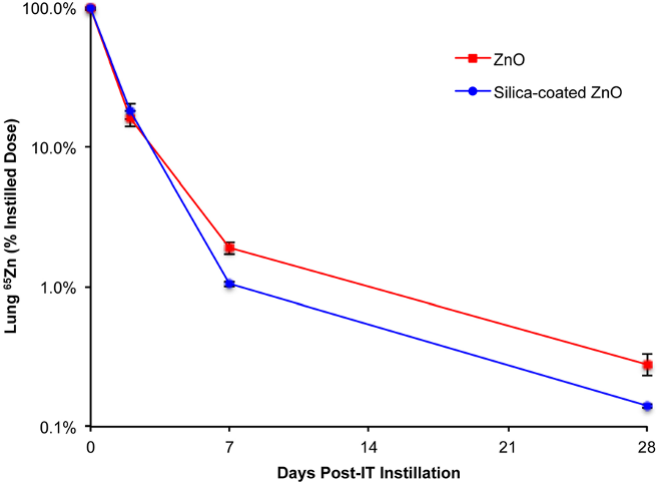
**Lung clearance of**^**65**^**Zn post-IT instillation of**^**65**^**ZnO and SiO**_**2**_**-coated**^**65**^**ZnO NPs.** The percentages of instilled ^65^Zn measured in the whole lungs are shown over a period of 28 days. The clearance of ^65^Zn was rapid with only 16-18% of dose remaining at 2 days. By day 7, only 1.1% (SiO_2_-coated ^65^ZnO NPs) and 1.9% (^65^ZnO NPs) were measured in the lungs. And by the end of experiment, ^65^Zn was nearly gone (less than 0.3% of dose). Although statistically higher levels of ^65^ZnO NPs than of SiO_2_-coated ^65^ZnO NPs remained in the lungs at 7 and 28 days, the graphs show nearly identical clearance kinetics. (n = 8 rats at 5 minutes, 2 days, and 7 days, n = 5 at 28 days).

Clearance of instilled uncoated or SiO_2_-coated ^65^ZnO NPs from the lungs is shown in Figure 3. Overall, both ^65^ZnO NPs and SiO_2_-coated ^65^ZnO NPs exhibited a biphasic clearance with a rapid initial phase (t_1/2_: ^65^ZnO = 0.3 hours; SiO_2_-coated ^65^ZnO = 0.2 hours) and a slower terminal phase (t_1/2_: ^65^ZnO = 42 hours; SiO_2_-coated ^65^ZnO = 29 hours). No significant difference was observed on the initial clearance between the two types of NPs. At 2 days, 18.1 ± 2.1% and 16.1 ± 2.0% remained in the lungs for the SiO_2_-coated and uncoated ^65^ZnO NPs, respectively. At 7 and 28 days post-IT instillation, we observed statistically significant but small (in magnitude) differences. At 28 days, only 0.14 ± 0.01% of SiO_2_-coated ^65^ZnO and 0.28 ± 0.05% of the uncoated ^65^ZnO NPs remained in the lungs.

**Figure 4:**
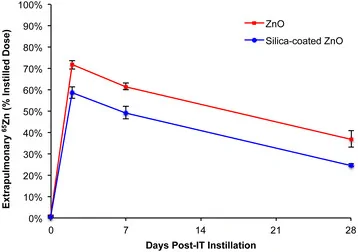
**Extrapulmonary distribution of**^**65**^**Zn post-IT instillation of**^**65**^**ZnO and SiO**_**2**_**-coated**^**65**^**ZnO NPs.** Data are % of instilled dose recovered in all secondary tissues examined. It included blood, thoracic lymph nodes, bone, bone marrow, skin, brain, skeletal muscle, testes, kidneys, heart, liver, and the gastrointestinal tract. There was a rapid absorption and accumulation of ^65^Zn in secondary tissues. At day 2, 59-72% of the dose was detected in extrapulmonary organs. Then, ^65^Zn levels decreased over time to 25-37% by day 28. Significantly more ^65^Zn was detected in secondary organs at all time points in rats instilled with uncoated ^65^ZnO NPs.

**Figure 5:**
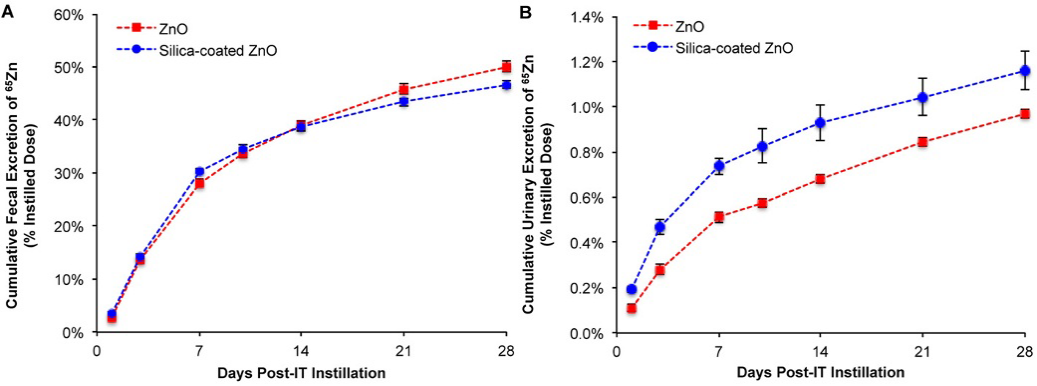
**Fecal and urinary excretion of**^**65**^**Zn post-IT instillation of**^**65**^**ZnO and SiO**_**2**_**-coated**^**65**^**ZnO NPs.** Data are estimated cumulative urinary or fecal excretion of ^65^Zn over 28 days. The predominant excretion pathway was via the feces. Approximately half of the instilled ^65^Zn was excreted in the feces in both groups over 28 days **(A)**. Only about 1% of the ^65^Zn dose was excreted in the urine **(B)**.

However, analyses of the selected extrapulmonary tissues showed significant differences (Figure 4). Even at the earliest time point of 5 minutes post-IT instillation, significantly more ^65^Zn was detected in the blood (0.47% vs. 0.25%) and heart (0.03% vs. 0.01%) of rats instilled with the uncoated ^65^ZnO NPs. These tissue differences became more pronounced at later time points. At 2 days post-IT instillation, more ^65^Zn from uncoated ^65^ZnO NPs translocated to the blood, skeletal muscle, kidneys, heart, liver and cecum than from SiO_2_-coated ^65^ZnO NPs (Table 1). At 7 and 28 days, the overall differences in the ^65^Zn contents in these tissues remained the same. As shown in Tables 2 and 3, significantly higher fractions of the ^65^Zn from uncoated ^65^ZnO NPs than from SiO_2_-coated ^65^ZnO NPs were found in the blood, skeletal muscle, heart, liver and skin. Interestingly, higher percentages of ^65^Zn dose from the SiO_2_-coated ^65^ZnO NPs were found in the thoracic lymph nodes and bone marrow (Tables 2 and 4). Radioactive ^65^Zn levels decreased from 2 to 28 days in all tissues except bone, where it increased for both types of NPs. Additionally, we found that the total recovered ^65^Zn in examined tissues, feces and urine was significantly higher in uncoated than SiO_2_-coated ^65^ZnO NPs (Tables 1, 2,3 and Figure 5). Since the thoracic lymph nodes had higher ^65^Zn in the latter group at all time points (Tables 1, 2 and 3), we speculate that the unaccounted radioactivity may have been in other lymph nodes as well as organs not analyzed such as adipose tissue, pancreas, adrenals, teeth, nails, tendons, and nasal tissues.

**Table 1:** **Tissue distribution of**^**65**^ **Zn at 2 days after intratracheal instillation of** ^**65**^ **ZnO or SiO**_**2**_ **-coated** ^**65**^ **ZnO NPs in rats**

	ZnO	SiO_2_-coated ZnO
	Mean ± SE	Mean ± SE
Lungs	16.08 ± 2.00	18.09 ± 2.17
Blood	2.54 ± 0.07*	2.22 ± 0.08
Lymph nodes	0.63 ± 0.23	0.49 ± 0.04
Bone marrow	3.37 ± 0.32	2.89 ± 0.15
Bone	9.59 ± 0.46	9.25 ± 0.29
Skin	11.30 ± 1.00	12.52 ± 0.77
Brain	0.17 ± 0.01	0.19 ± 0.01 #
Skeletal muscle	14.22 ± 0.77*	6.39 ± 2.44
Testes	0.84 ± 0.05	0.78 ± 0.04
Kidneys	2.05 ± 0.07*	1.74 ± 0.03
Spleen	0.50 ± 0.02	0.44 ± 0.02
Heart	0.47 ± 0.04*	0.36 ± 0.01
Liver	12.23 ± 0.24*	9.88 ± 0.38
Stomach	1.01 ± 0.14	0.78 ± 0.02
Small intestine	7.37 ± 0.32	6.89 ± 0.20
Large intestine	2.08 ± 0.21	1.91 ± 0.12
Cecum	3.42 ± 0.22*	2.35 ± 0.25
Total recovered	87.78 ± 2.35*	76.78 ± 2.84

Data are mean ± SE% instilled dose, n = 8/group.

Total recovered = sum of ^65^Zn in analyzed organs, feces and urine.

*P < 0.05, ZnO > SiO_2_-coated ZnO.

# P <0.05, SiO_2_-coated ZnO > ZnO.

**Table 2:** **Tissue distribution of** ^**65**^ **Zn at 7 days after intratracheal instillation of** ^**65**^ **ZnO or SiO**_**2**_ **-coated** ^**65**^ **ZnO NPs in rats**

	^65^ZnO	SiO_2_-coated^65^ZnO
	Mean ± SE	Mean ± SE
Lungs	1.90 ± 0.18	1.05 ± 0.04
Blood	2.13 ± 0.05*	1.79 ± 0.07
Lymph nodes	0.18 ± 0.02	0.31 ± 0.05 #
Bone marrow	3.70 ± 0.28	3.38 ± 0.16
Bone	12.12 ± 0.53	12.21 ± 0.84
Skin	10.02 ± 0.49	10.55 ± 0.80
Brain	0.25 ± 0.01	0.27 ± 0.01
Skeletal muscle	19.81 ± 0.84*	8.34 ± 3.45
Testes	1.27 ± 0.06	1.23 ± 0.03
Kidneys	0.75 ± 0.03	0.69 ± 0.02
Spleen	0.21 ± 0.01	0.20 ± 0.01
Heart	0.27 ± 0.01*	0.23 ± 0.01
Liver	5.80 ± 0.13*	5.19 ± 0.25
Stomach	0.66 ± 0.02	0.66 ± 0.03
Small intestine	2.83 ± 0.10	2.53 ± 0.12
Large intestine	0.84 ± 0.07	0.83 ± 0.05
Cecum	1.15 ± 0.06	1.04 ± 0.09
Total recovered	81.33 ± 6.51*	69.91 ± 3.33

Data are mean ± SE% instilled dose, n = 8/group.

Total recovered = sum of ^65^Zn in analyzed organs, feces and urine.

*P < 0.05, ZnO > SiO_2_-coated ZnO.

# P <0.05, SiO_2_-coated ZnO > ZnO.

**Table 3:** **Tissue distribution of** ^**65**^ **Zn at 28 days after intratracheal instillation of** ^**65**^ **ZnO or SiO**_**2**_ **-coated** ^**65**^ **ZnO NPs in rats**

	^65^ZnO	SiO_2_-coated^65^ZnO
	Mean ± SE	Mean ± SE
Lungs	0.28 ± 0.05*	0.14 ± 0.01
Blood	0.79 ± 0.05*	0.61 ± 0.02
Lymph nodes	0.03 ± 0.005	0.12 ± 0.01 #
Bone marrow	2.30 ± 0.12	3.33 ± 0.15 #
Bone	12.40 ± 0.36	13.59 ± 0.52
Skin	4.72 ± 0.60*	3.26 ± 0.13
Brain	0.18 ± 0.02	0.15 ± 0.01
Skeletal muscle	13.27 ± 3.02*	0.66 ± 0.04
Testes	0.49 ± 0.03	0.46 ± 0.03
Kidneys	0.19 ± 0.01	0.17 ± 0.005
Spleen	0.05 ± 0.005	0.04 ± 0.003
Heart	0.07 ± 0.004*	0.06 ± 0.002
Liver	1.32 ± 0.09	1.14 ± 0.02
Stomach	0.20 ± 0.01	0.20 ± 0.01
Small intestine	0.63 ± 0.05	0.55 ± 0.01
Large intestine	0.19 ± 0.02	0.25 ± 0.01 #
Cecum	0.23 ± 0.02	0.24 ± 0.01
Total recovered	88.20 ± 4.36*	72.81 ± 0.53

Data are mean ± SE% instilled dose, n = 5/group.

Total recovered = sum of ^65^Zn in analyzed organs, feces and urine.

*P < 0.05, ZnO > SiO_2_-coated ZnO.

# P <0.05, SiO_2_-coated ZnO > ZnO.

**Table 4:** **Distribution of** ^**65**^ **Zn 7 days after gavage administration of** ^**65**^ **ZnO or SiO**_**2**_ **-coated** ^**65**^ **ZnO NPs in rats**

	^65^ZnO	SiO_2_-coated^65^ZnO
	Mean ± SE	Mean ± SE
Lungs	0.04 ± 0.01	0.06 ± 0.01
Blood	0.23 ± 0.03	0.22 ± 0.04
Lymph nodes	0.02 ± 0.003	0.06 ± 0.01 #
Bone marrow	0.49 ± 0.06	0.47 ± 0.09
Bone	1.62 ± 0.26	2.20 ± 0.45
Skin	1.13 ± 0.14	1.77 ± 0.29
Brain	0.03 ± 0.002	0.04 ± 0.005
Skeletal muscle	2.45 ± 0.36*	0.20 ± 0.03
Testes	0.14 ± 0.01	0.19 ± 0.03
Kidneys	0.08 ± 0.01	0.09 ± 0.01
Spleen	0.02 ± 0.004	0.02 ± 0.004
Heart	0.03 ± 0.003	0.02 ± 0.004
Liver	0.58 ± 0.07	0.71 ± 0.09
Stomach	0.07 ± 0.01	0.09 ± 0.01
Small intestine	0.25 ± 0.03	0.36 ± 0.05
Large intestine	0.09 ± 0.02	0.11 ± 0.01
Cecum	0.13 ± 0.02	0.12 ± 0.02
Total recovered	100.59 ± 2.56*	83.40 ± 2.42

Data are mean ± SE% gavaged dose, n = 5/group.

Total recovered = sum of ^65^Zn in analyzed organs, feces and urine.

*P < 0.05, ZnO > SiO_2_-coated ZnO.

# P <0.05, SiO_2_-coated ZnO > ZnO.

Urinary excretion of ^65^Zn was much lower than fecal excretion in both groups. The urinary excretion of ^65^Zn in rats instilled with SiO_2_-coated ^65^ZnO NPs was significantly higher than in those instilled with uncoated ^65^ZnO NPs (Figure 5B). Although the fecal excretion rates appeared similar, slightly but significantly more ^65^Zn (50.04 ± 0.96% vs. 46.68 ± 0.76%) was eliminated via the feces over 28 days in rats instilled with uncoated ^65^ZnO NPs (Figure 5A).

### Pharmacokinetics of gavaged uncoated or SiO_2_-coated ^65^ZnO NPs

**Figure 6:**
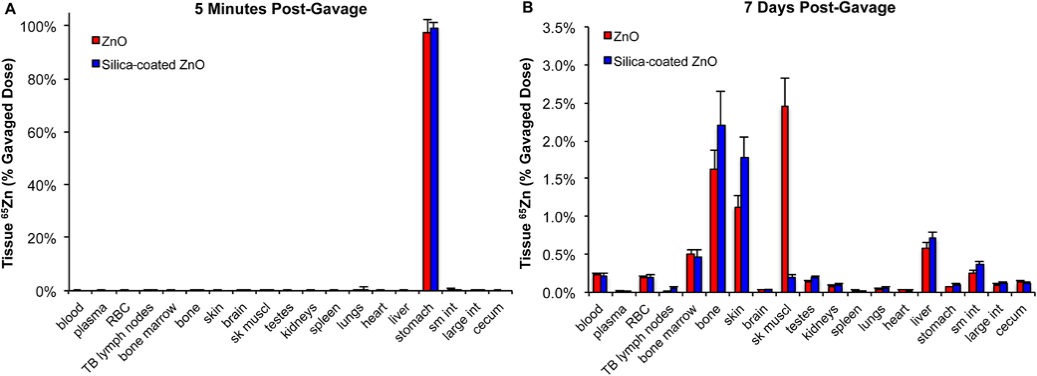
**Tissue distribution of**^**65**^**Zn post-gavage of**^**65**^**ZnO and SiO**_**2**_**-coated**^**65**^**ZnO NPs.** Data are % dose of administered ^65^Zn in different organs. **(A)** At 5 minutes post-gavage, the ^65^Zn levels in tissues other than the gastrointestinal tract were much lower (0.3% for uncoated, 0.05% for coated ^65^ZnO NPs). **(B)** At day 7, significantly more ^65^Zn was absorbed and retained in non-GIT tissues (6.9% for uncoated, 6.0% for coated ^65^ZnO NPs). Significantly more ^65^Zn was measured in skeletal muscle in rat gavaged with uncoated versus coated ^65^ZnO NPs. (Note: RBC: red blood cell; sk muscl: skeletal muscle; sm int: small intestine: large int: large intestine).

**Figure 7:**
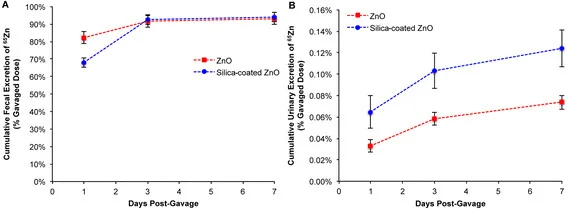
**Fecal and urinary excretion of**^**65**^**Zn post-gavage of**^**65**^**ZnO and SiO**_**2**_**-coated**^**65**^**ZnO NPs.** Data are estimated cumulative urinary or fecal excretion of ^65^Zn over 7 days. Similar to the IT-instilled groups, the predominant excretion pathway was via the feces. Ninety five % of the instilled ^65^Zn was excreted in both groups by day 7 **(A)**. Only 0.1% of the ^65^Zn dose was excreted in the urine **(B)**.

Absorption of ^65^Zn from the gut was studied at 5 minutes and 7 days post-gavage of uncoated or SiO_2_-coated ^65^ZnO NPs. Nearly 100% of the dose was recovered at 5 minutes in the stomach for both types of NPs (Figure 6A). The ^65^Zn levels in tissues other than the gastrointestinal tract were much lower (0.3% for uncoated, 0.05% for coated ^65^ZnO NPs). However, significantly higher percentages of total dose were still detected in the blood, bone marrow, skin, testes, kidneys, spleen and liver in rats instilled with uncoated ^65^ZnO NPs (data not shown). After 7 days, low levels of ^65^Zn from both types of NPs (<1% original dose) were measured in all organs except the bone, skeletal muscle and skin (Figure 6B, Table 4). Higher levels of ^65^Zn were observed in the skeletal muscle from uncoated than from coated ^65^ZnO NPs at this time point (Table 4). However, similar to the IT-instillation data, the thoracic lymph nodes retained more ^65^Zn from the SiO_2_-coated than the uncoated ^65^ZnO NPs. Urinary excretion of ^65^Zn was also much lower than fecal excretion post-gavage. The urinary excretion of ^65^Zn in rats gavaged with SiO_2_-coated ^65^ZnO NPs was significantly higher than in rats gavaged with uncoated ^65^ZnO NPs (Figure 7B). The fecal excretion in the gavaged rats was higher than in IT-instilled rats. Despite a significant difference in fecal excretion during the first day post-gavage, nearly 95% of the dose for both types of NPs was excreted in the feces by day 7 (Figure 7A).

## Discussion

Nanoparticles can be released into the workplace environment during production and handling of nanomaterials [42]. For example, studies have shown that ZnO NPs were released during an abrasion test of commercially available two-pack polyurethane coatings with ZnO NPs [43]. This suggests the likelihood of emission of NPs during activities related to handling of nano-enabled products. In this study we describe the acute pulmonary responses to ZnO NPs and the pharmacokinetics of Zn from ZnO or SiO_2_-coated ZnO NPs in male Wistar Han rats. To track Zn for biokinetic studies in rats, we neutron activated the NPs to change the stable element ^64^Zn into radioactive ^65^Zn, suitable for detection over long-term studies. The agglomerate size and zeta potential in water suspension were similar to those of pristine ZnO NPs. Using these radioactive NPs, we evaluated the influence of an amorphous silica coating on the clearance, bioavailability and excretion of ^65^Zn following intratracheal instillation and gavage of ^65^ZnO and Si-coated ^65^ZnO NPs. We have shown previously that the hermetic encapsulation of ZnO NPs with a thin layer of amorphous SiO_2_ reduces the dissolution of Zn^2+^ ions in biological media, DNA damage in vitro [17] and cellular toxicity [36]. Since the SiO_2_ coating does not affect the core ZnO NP optoelectronic properties, these coatings may be employed in sunscreens and UV filters. This could be a strategy to reduce ZnO toxicity while maintaining the intended performance of ZnO NPs.

Intratracheal instillation differs from inhalation exposure in terms of particle distribution, dose rate, clearance, NP agglomerate surface properties, and pattern of injury [44],[45]. A study by Baisch et al. reported a higher inflammatory response following intratracheal instillation compared to whole body inhalation for single and repeated exposures of titanium dioxide NPs when deposited doses were held constant [46]. Although IT instillation does not directly model inhalation exposure, it is a reliable method for administering a precise dose to the lungs for biokinetic studies. We hypothesized that silica coating may alter zinc-induced lung injury and inflammation by reducing the available zinc ions based on our previous data [17]. We have also shown that pulmonary toxicity in rats exposed to nanoceria via inhalation was reduced when exposed to the same nanoceria with amorphous SiO_2_ coating. Surprisingly, the in vivo lung responses in the present study showed the opposite. That amorphous silica can cause injury and inflammation when inhaled at high doses has been shown in several previous studies [47]–[51]. However, it has also been shown that the lung injury and inflammatory responses to amorphous silica are transient [27]. In this study, SiO_2_-coated ZnO NPs induce more lung injury/inflammation than uncoated ZnO, even at a low dose at which uncoated ZnO had no effects. Considering that the effective density of ZnO NPs is reduced by silica coating (ZnO: 5.6 g/cm^3^ vs. SiO_2_-coated ZnO: estimated 4.1 g/cm^3^), it is possible that the coated particle number concentration is higher for an equivalent mass of NP. It is also likely that the silica coating elicits more inflammation than the ZnO NPs. Silica may act in concert with dissolved Zn ions, causing more lung injury. Furthermore, surface coating with amorphous silica also changed the zeta-potential of ZnO NPs from positive (23.0 ± 0.4 mV, uncoated ZnO NPs) to negative (−16.2 ± 1.2 mV, SiO_2_-coated ZnO NPs), decreasing the likelihood of agglomeration and sedimentation of SiO_2_-coated NP suspension in aqueous systems. The reduced NP agglomeration of the SiO_2_-coated ZnO NPs may increase the available NP surface area that may facilitate biointeractions with lung cells and thus induces a higher toxic/inflammatory response. It has also been reported that surface charge may influence the lung translocation rates of NPs [52]. For example, the adsorption of endogenous proteins like albumin to the surface of charged NPs increases their hydrodynamic diameter and alters their translocation rate [53]. It was also showed that NPs with zwitterionic cysteine and polar PEG ligands on the surface cause their rapid translocation to the mediastinal lymph nodes. Additionally, a higher surface charge density has been shown to cause an increased adsorption of proteins on NPs [54] while zwitterionic or neutral organic coatings have been shown to prevent adsorption of serum proteins [18]. A recent study also showed that nanoparticle protein corona can alter their uptake by macrophages [55].

Our results demonstrate that ZnO and SiO_2_-coated ZnO NPs are both cleared rapidly and completely from the lungs by 28 days after IT instillation. In the lungs, NPs may be cleared via different pathways. They may be cleared by dissolution before or after alveolar macrophage uptake, by phagocytic cells in the lymph nodes, or by translocation across the alveolar epithelium into the blood circulation [56]. Since ZnO NPs have been shown to dissolve in culture medium and in endosomes [57], it is not surprising that lung clearance of ^65^ZnO NPs was rapid compared to that of poorly soluble NPs of cerium oxide [58] and titanium dioxide [59]. The clearance of radioactive ^65^Zn from the lungs includes translocation of the NPs themselves as well as dissolution of ^65^ZnO which is an important clearance mechanism [60]. As shown previously, the silica coating reduced the dissolution of ZnO NPs in culture medium [17], suggesting that dissolution and clearance in vivo may also be reduced. However, the silica coating appeared to very modestly but significantly enhance the amount of cleared ^65^Zn at day 7 and 28. The significance of this observation needs further investigation.

Despite similar clearance from the lungs over 28 days, translocation of ^65^Zn from uncoated ZnO NPs is significantly higher than from coated ZnO NPs in some of the examined extrapulmonary tissues, especially skeletal muscle. In these extrapulmonary tissues, the measured ^65^Zn is more likely to be dissolved Zn, rather than intact ^65^ZnO. The amount of ^65^Zn was greatest in the skeletal muscle, liver, skin, and bone from both particle types. The selective retention of ^65^Zn into those tissues might be explained, in part, by the fact that 85% of the total body zinc is present in skeletal muscle and bone [61]. There was clearance of ^65^Zn from most of the extrapulmonary tissues we examined over time (day 2 to day 28), except in bone where ^65^Zn levels increased. The skin and skeletal muscle exhibited faster clearance with coated than with uncoated NPs. ^65^Zn from both particle types was largely excreted in the feces, presumably via pancreato-biliary secretion, and to a lesser extent via mucociliary clearance of instilled NPs [62]. A study investigating the pharmacokinetic behavior of inhaled iridium NPs showed that they accumulated in soft connective tissue (2%) and bone, including bone marrow (5%) [63].

Although this study indicates that the SiO_2_ coating modestly reduces the translocation of ^65^Zn to the blood, skin, kidneys, heart, liver and skeletal muscle, it is unclear whether the SiO_2_-coated ZnO NPs dissolve at a different rate in vivo, and whether ^65^Zn is in particulate or ionic form when it reaches the circulation and bone. ZnO NPs have been shown to rapidly dissolve under acidic conditions (pH 4.5) and are more likely to remain intact around neutral pHs [64]. It is likely that the ZnO NPs entering phagolysosomal compartments of alveolar macrophages or neutrophils may encounter conditions favorable for dissolution. Our previous study suggested that the SiO_2_ coating is stable in vitro and exhibits low dissolution in biological media (<8% over 24 hours) [17]. Thus, it is possible that the SiO_2_-coated NPs remain in particulate form for a longer period of time. There are data showing that translocation of gold, silver, TiO_2_, polystyrene and carbon particles in the size range of 5–100 nm crossing the air-blood barrier and reaching blood circulation and extrapulmonary organs can occur [65]–[71].

The SiO_2_ coating significantly increased the levels of ^65^Zn in the bone and bone marrow (Table 3). We note that zinc is essential to the development and maintenance of bone. Zinc is known to play a major role in bone growth in mammals [72], and is required for protein synthesis in osteoblasts [73]. It can also inhibit the development of osteoclasts from bone marrow cells, thereby reducing bone resorption and bone growth [74],[75]. Radioactive ^65^Zn from uncoated and coated ^65^ZnO NPs also translocated to the skin, skeletal muscle, liver, heart, small intestine, testes, and brain (but to a lesser extent than the bone and bone marrow). It is important to note that of the 16 extrapulmonary tissues examined at 28 days after IT instillation, 4 had a higher ^65^Zn content from uncoated ZnO than coated ZnO (blood, skin, skeletal muscle and heart) (Table 3). This suggests that amorphous silica coating of NPs may reduce Zn retention and its potential toxicity when accumulated at high levels in those organs. Whether coating modifications like the use of thicker or different coatings can further reduce Zn bioavailability warrants further investigation. There was significantly more ^65^Zn from SiO_2_-coated ZnO excreted in the urine, which was more likely the ionic form of Zn.

The oral exposure to ZnO NPs is relevant from an environmental health perspective. ZnO is widely used as a nutritional supplement and as a food additive [76]. Because it is an essential trace element, zinc is routinely added to animal food products and fertilizer [75]. Due to its antimicrobial properties, there is increasing interest in adding ZnO to polymers in food packaging and preservative films to prevent bacterial growth [77]. It is possible that ZnO in sunscreens, ointments, and other cosmetics can be accidentally ingested, especially by children. The biokinetic behavior of NPs in the gastrointestinal tract may be influenced by particle surface charge. Positively charged particles are attracted to negatively charged mucus, while negatively charged particles directly contact epithelial cell surfaces [78]. A study by Paek et al. investigating the effect of surface charge on the biokinetics of Zn over 4 hours after oral administration of ZnO NPs showed that negatively charged NPs were absorbed more than positively charged ZnO NPs [79]. However, no effect on tissue distribution was observed. This is in contrast to our findings at 7 days post-gavage when coating of ZnO NPs with amorphous SiO_2_ (with negative zeta potential) increased the retention in thoracic lymph nodes compared to uncoated ZnO NPs (with positive zeta potential). Our study also showed that low levels of ^65^Zn were retained in the blood, skeletal muscle, bone and skin from both coated and uncoated ^65^ZnO NPs (Table 4). Most of the gavaged dose (over 90%) was excreted in the feces by day 3 indicating a rapid clearance of ZnO NPs, consistent with previous reports. Another study reported the pharmacokinetics of ZnO NPs (125, 250 and 500 mg/kg) after a single and repeated dose oral administration (90-day) [80]. They found that plasma Zn concentration significantly increased in a dose-dependent manner, but significantly decreased within 24 hours post-oral administration, suggesting that the systemic clearance of ZnO NPs is rapid even at these high doses. In another study, Baek et al. examined the pharmacokinetics of 20 nm and 70 nm citrate-modified ZnO NPs at doses of 50, 300 and 2000 mg/kg [81]. Similar to our results, they showed that ZnO NPs were not readily absorbed into the bloodstream after single-dose oral administration. The tissue distributions of Zn from both 20 nm and 70 nm ZnO NPs were similar and mainly to the liver, lung and kidneys. The study also reported predominant excretion of Zn in the feces, with smaller 20 nm particles being cleared more rapidly than the 70 nm NPs.

In summary, the results presented here show that uncoated ^65^Zn NPs resulted in higher levels of ^65^Zn in multiple organs following intratracheal instillation or gavage, particularly in skeletal muscle. This suggests that coating with amorphous silica can reduce tissue Zn concentration and its potential toxicity. Interestingly, the bioavailability of Zn from SiO_2_-coated ^65^ZnO was higher in thoracic lymph nodes and bone. Additionally, the excretion of ^65^Zn was higher from SiO_2_-coated ^65^ZnO NPs from both routes suggesting enhanced hepatobiliary excretion. Our data indicate that silica coating alters the pharmacokinetic behavior of ZnO NPs, but the effect was not as dramatic as anticipated. With increasing trends in physicochemical modifications of NPs for special applications, it is necessary to understand their influence on the fate, metabolism and toxicity of these nanoparticles.

## Conclusions

We examined the influence of a 4.5 nm SiO_2_ coating on ZnO NPs on the ^65^Zn pharmacokinetics following IT instillation and gavage of neutron activated NPs. The SiO_2_ coating does not affect the clearance of ^65^Zn from the lungs. However, the extrapulmonary translocation and distribution of ^65^Zn from coated versus uncoated ^65^ZnO NPs were significantly altered in some tissues. The SiO_2_ coating resulted in lower translocation of instilled ^65^Zn to the skeletal muscle, skin and heart. The SiO_2_ coating also reduced ^65^Zn translocation to skeletal muscle post-gavage. For both routes of administration, the SiO_2_ coating enhanced the transport of ^65^Zn to the thoracic lymph nodes.

## Methods

### Synthesis of ZnO and SiO_2_-coated ZnO NPs

The synthesis of these NPs was reported in detail elsewhere [17]. In brief, uncoated and SiO_2_-coated ZnO particles were synthesized by flame spray pyrolysis (FSP) of zinc naphthenate (Sigma-Aldrich, St. Louis, MO, USA) dissolved in ethanol (Sigma-Aldrich) at a precursor molarity of 0.5 M. The precursor solution was fed through a stainless steel capillary at 5 ml/min, dispersed by 5 L/min O_2_ (purity > 99%, pressure drop at nozzle tip: p_drop_ = 2 bar) (Air Gas, Berwyn, PA, USA) and combusted. A premixed methane-oxygen (1.5 L/min, 3.2 L/min) supporting flame was used to ignite the spray. Oxygen (Air Gas, purity > 99%) sheath gas was used at 40 L/min. Core particles were coated in-flight by the swirl-injection of hexamethyldisiloxane (HMDSO) (Sigma Aldrich) through a torus ring with 16 jets at an injection height of 200 mm above the FSP burner. A total gas flow of 16 L/min, consisting of N_2_ carrying HMDSO vapor and pure N_2_, was injected through the torus ring jets. HMDSO vapor was obtained by bubbling N_2_ gas through liquid HMDSO (500 ml), maintained at a controlled temperature using a temperature-controlled water bath.

### Characterization of ZnO and SiO_2_-coated ZnO NPs

The morphology of these NPs was examined by electron microscopy. Uncoated and SiO_2_-coated ZnO NPs were dispersed in ethanol at a concentration of 1 mg/ml in 50 ml polyethylene conical tubes and sonicated at 246 J/ml (Branson Sonifier S-450A, Swedesboro, NJ, USA). The samples were deposited onto lacey carbon TEM grids. All grids were imaged with a JEOL 2100. The primary particle size was determined by X-ray diffraction (XRD). XRD patterns for uncoated ZnO and SiO_2_-coated ZnO NPs were obtained using a Scintag XDS2000 powder diffractometer (Cu Kα, λ = 0.154 nm, 40 kV, 40 mA, stepsize = 0.02°). One hundred mg of each sample was placed onto the diffractometer stage and analyzed from a range of 2θ = 20-70°. Major diffraction peaks were identified using the Inorganic Crystal Structure Database (ICSD) for wurtzite (ZnO) crystals. The crystal size was determined by applying the Debye-Scherrer Shape Equation to the Gaussian fit of the major diffraction peak. The specific surface area was obtained using the Brunauer-Emmet-Teller (BET) method. The samples were degassed in N_2_ for at least 1 hour at 150°C before obtaining five-point N_2_ adsorption at 77 K (Micrometrics Tristar 3000, Norcross, GA, USA).

### Neutron activation of NPs

The NPs with and without the SiO_2_ coating were neutron-activated at the Massachusetts Institute of Technology (MIT) Nuclear Reactor Laboratory (Cambridge, MA). Samples were irradiated with a thermal neutron flux of 5 × 10^13^ n/cm^2^s for 120 hours. The resulting ^65^Zn radioisotope has a half-life of 244.3 days and a primary gamma energy peak of 1115 keV. The relative specific activities for ^65^Zn were 37.7 ± 5.0 kBq/mg for SiO_2_-coated ^65^ZnO and 41.7 ± 7.2 kBq/mg for ^65^ZnO NPs.

### Preparation and characterization of ZnO and SiO_2_-coated ZnO nanoparticle suspensions

Uncoated and SiO_2_-coated ZnO NPs were dispersed using a protocol previously described [82],[36]. The NPs were dispersed in deionized water at a concentration of 0.66 mg/ml (IT) or 10 mg/ml (gavage). Sonication was performed in deionized water to minimize the formation of reactive oxygen species. Samples were thoroughly mixed immediately prior to instillation. Dispersions of NPs were analyzed for hydrodynamic diameter (d_H_), polydispersity index (PdI), and zeta potential (ζ) by DLS using a Zetasizer Nano-ZS (Malvern Instruments, Worcestershire, UK).

### Animals

The protocols used in this study were approved by the Harvard Medical Area Animal Care and Use Committee. Nine-week-old male Wistar Han rats were purchased from Charles River Laboratories (Wilmington, MA). Rats were housed in pairs in polypropylene cages and allowed to acclimate for 1 week before the studies were initiated. Rats were maintained on a 12-hour light/dark cycle. Food and water were provided *ad libitum*.

### Pulmonary responses – Bronchoalveolar lavage and analyses

This experiment was performed to determine pulmonary responses to instilled NPs. A group of rats (mean wt. 264 ± 15 g) was intratracheally instilled with either an uncoated ZnO or SiO_2_ -coated ZnO NP suspension at a 0, 0.2 or 1.0 mg/kg dose. The particle suspensions were delivered to the lungs through the trachea in a volume of 1.5 ml/kg. Twenty-four hours later, rats were euthanized via exsanguination with a cut in the abdominal aorta while under anesthesia. The trachea was exposed and cannulated. The lungs were then lavaged 12 times, with 3 ml of 0.9% sterile PBS, without calcium and magnesium ions. The cells of all washes were separated from the supernatant by centrifugation (350 × g at 4°C for 10 min). Total cell count and hemoglobin measurements were made from the cell pellets. After staining the cells, a differential cell count was performed. The supernatant of the two first washes was clarified via centrifugation (14,500 × g at 4°C for 30 min), and used for standard spectrophotometric assays for lactate dehydrogenase (LDH), myeloperoxidase (MPO) and albumin.

### Pharmacokinetics of ^65^Zn

The mean weight of rats at the start of the experiment was 285 ± 3 g. Two groups of rats (29 rats/NP) were intratracheally instilled with ^65^ZnO NPs or with SiO_2_-coated ^65^ZnO NPs at a 1 mg/kg dose (1.5 ml/kg, 0.66 mg/ml). Rats were placed in metabolic cages containing food and water, as previously described. Twenty four-hour samples of feces and urine were collected at selected time points (0–24 hours, 2–3 days, 6–7 days, 9–10 days, 13–14 days, 20–21 days, and 27–28 days post-IT instillation). Fecal/urine collection was accomplished by placing each rat in individual metabolic cage containing food and water during each 24-hour period. All samples were analyzed for total ^65^Zn activity, and expressed as % of instilled ^65^Zn dose. Fecal and urine clearance curves were generated and were used to estimate the daily cumulative excretion. Groups of 8 rats were humanely sacrificed at 5 minutes, 2 days, 7 days, and 5 rats/group at 28 days. Therefore, the number of collected fecal/urine samples decreased over time.

Another cohort of 20 rats was dosed with ^65^ZnO (n = 10) or SiO_2_-coated ^65^ZnO (n = 10) by gavage at a 5 mg/kg dose (0.5 ml/kg, 10 mg/ml). One group of 5 rats was humanely sacrificed at 5 minutes and immediately dissected. Another group of 5 rats was individually placed in metabolic cages, as previously described, and 24-hour samples of urine and feces were collected at 0–1 day, 2–3 days, and 6–7 days post-gavage. The remaining rats were sacrificed at 7 days.

At each endpoint, rats were euthanized and dissected, and the whole brain, spleen, kidneys, heart, liver, lungs, GI tract, testes, thoracic lymph nodes, blood (10 ml, separated into plasma and RBC), bone marrow (from femoral bones), bone (both femurs), skin (2 × 3 inches), and skeletal muscle (from 4 sites) were collected. The ^65^Zn radioactivity present in each sample was measured with a WIZARD Gamma Counter (PerkinElmer, Inc., Waltham, MA). The number of disintegrations per minute was determined from the counts per minute and the counting efficiency. The efficiency of the gamma counter was derived from counting multiple aliquots of NP samples and relating them to the specific activities measured at Massachusetts Institute of Technology Nuclear Reactor. We estimated that the counter had an efficiency of ~52%. The ^65^Zn radioactivity was expressed as kBq/g tissue and the percentage of administered dose in each organ. All radioactivity data were adjusted for physical decay over the entire observation period. The radioactivity in organs and tissues not measured in their entirety was estimated as a percentage of total body weight as: skeletal muscle, 40%; bone marrow, 3.2%; peripheral blood, 7%; skin, 19%; and bone, 6% [83],[84]. Based on the ^65^Zn specific activity (kBq/mg NP) and tissue ^65^Zn concentration, the amount of Zn derived from each NP was calculated for each tissue examined (ng Zn/g tissue).

### Statistical analyses

Differences in the ^65^Zn tissue distribution and in cellular and biochemical parameters measured in bronchoalveolar lavage between groups were analyzed using multivariate analysis of variance (MANOVA) with REGWQ (Ryan-Einot-Gabriel-Welch based on range) and Tukey post hoc tests using SAS Statistical Analysis software (SAS Institute, Cary, NC). The lung clearance half-life was estimated by a two-phase estimation by a biexponential model using the R Program v. 3.1.0 [85].

## Authors’ original submitted files for images

Below are the links to the authors’ original submitted files for images. Authors’ original file for figure 1 Authors’ original file for figure 2 Authors’ original file for figure 3 Authors’ original file for figure 4 Authors’ original file for figure 5 Authors’ original file for figure 6 Authors’ original file for figure 7
